# Translating Evidence Into Practice via Social Media: A Mixed-Methods Study

**DOI:** 10.2196/jmir.4763

**Published:** 2015-10-26

**Authors:** Stephen Maloney, Jacqueline Tunnecliff, Prue Morgan, Jamie E Gaida, Lyn Clearihan, Sivalal Sadasivan, David Davies, Shankar Ganesh, Patitapaban Mohanty, John Weiner, John Reynolds, Dragan Ilic

**Affiliations:** ^1^ Monash University Frankston Australia; ^2^ University of Canberra The University of Canberra Research Institute for Sport and Exercise (UCRISE) Canberra Australia; ^3^ University of Canberra Discipline of Physiotherapy Canberra Australia; ^4^ Monash University Sunway Malaysia; ^5^ University of Warwick Coventry United Kingdom; ^6^ Swami Vivekanand National Institute of Rehabilitation Training and Research (SVNIRTAR) Cuttack India; ^7^ Monash University Melbourne Australia

**Keywords:** social media, medical informatics, evidence-based practice, e-learning

## Abstract

**Background:**

Approximately 80% of research evidence relevant to clinical practice never reaches the clinicians delivering patient care. A key barrier for the translation of evidence into practice is the limited time and skills clinicians have to find and appraise emerging evidence. Social media may provide a bridge between health researchers and health service providers.

**Objective:**

The aim of this study was to determine the efficacy of social media as an educational medium to effectively translate emerging research evidence into clinical practice.

**Methods:**

The study used a mixed-methods approach. Evidence-based practice points were delivered via social media platforms. The primary outcomes of attitude, knowledge, and behavior change were assessed using a preintervention/postintervention evaluation, with qualitative data gathered to contextualize the findings.

**Results:**

Data were obtained from 317 clinicians from multiple health disciplines, predominantly from the United Kingdom, Australia, the United States, India, and Malaysia. The participants reported an overall improvement in attitudes toward social media for professional development (*P*<.001). The knowledge evaluation demonstrated a significant increase in knowledge after the training (*P*<.001). The majority of respondents (136/194, 70.1%) indicated that the education they had received via social media had changed the way they practice, or intended to practice. Similarly, a large proportion of respondents (135/193, 69.9%) indicated that the education they had received via social media had increased their use of research evidence within their clinical practice.

**Conclusions:**

Social media may be an effective educational medium for improving knowledge of health professionals, fostering their use of research evidence, and changing their clinical behaviors by translating new research evidence into clinical practice.

## Introduction

Greater accessibility to the Internet and mobile technologies has seen unprecedented opportunities for populations to access health information, and for health professionals to access new knowledge. In 2012, Facebook reported that it was hosting over one billion users, or 15% of the world’s population [1] and Twitter announced its users were sending 500 million tweets per day [2]. The possibilities this presents in health care for increasing the movement of knowledge from "bench to bedside" is however still relatively unexplored. It is currently estimated that 80% of research evidence relevant to clinical practice never reaches clinicians delivering patient care [3]. One of the key barriers for the translation of evidence from demonstrated benefit into practice is the limited time available for clinicians to search for, and appraise, emerging evidence [4]. This raises the issue of whether social media could provide a bridge between health researchers and health service providers, allowing researchers to directly message peer-reviewed findings to clinicians, and for clinicians to receive the messages on their Web-enabled devices at the point of care [5,6].

In a health care context, one definition of social media is a “collection of Web-based technologies that share a user-focused approach to design and functionality, where users can actively participate in content creation and editing through open collaboration between members of communities of practice” [7]. Social media, by overcoming barriers to engagement associated with geography, can expand community-of-practice networks, connecting practitioners irrespective of whether they are rural, remote, or metropolitan based. The global reach of the Internet facilitates a sharing and evolution of national and global perspectives. Leading global and national health authorities, journals, research centers, health professional education societies, universities, and even hospitals now have an active social media presence, which indicates the increasing recognition of the importance of a social media presence among leaders and governing bodies of health professional groups. Bergl et al have demonstrated the use of Twitter feeds to advance an internal medicine residency program’s educational mission, where Twitter enhanced the residents' overall education in residency [8]. The Cochrane Collaboration registered its first systematic review title on social media for clinical excellence in 2014. Arguably, social media has developed a "formal," professional side to its personality.

Although intuitively there seems to be mutual benefits to be gained from connecting clinicians directly with researchers, the issue has always been how do we create and facilitate effective connections? Social media would appear to offer this opportunity, as it becomes an acceptable medium between health researchers and clinicians in translating emerging evidence into practice [9]. However, in spite of growing research related to the pedagogical value of the media from an educational perspective and the role of blended learning modalities versus traditional formats for knowledge translation [10-14], the literature on the impact of social media on education has largely centered on opinions and arguments around culture change and aspects of the learning experience [7,15-17]. A systematic review exploring the uses, benefits, and limitations of social media for health communication was conducted by Moorhead et al [15]. Their review identified three overarching benefits of social media: increased interaction with others, more available shared and tailored information, and increased accessibility and widening access to health information. However, the vast majority of studies identified within their review were of a poor quality, largely due to the limitations of the methodologies used and the descriptive nature of the studies. Their review also identified the need to progress toward measuring the effectiveness of social media for knowledge translation. A further review conducted by Cheston et al exploring the use of social media in medical education identified only four studies that used an examination to assess knowledge change [7]. Of those studies, only one utilized a preintervention/postintervention design with data taken from the same participants, with empathy as their primary outcome of interest [18]. As yet, no published studies have reported an empirical evaluation of the impact and role of social media on translation of health research into practice.

We have thus undertaken a study that aims to determine the efficacy of social media as an educational medium to effectively translate emerging research evidence into clinical practice, through changing clinician knowledge and behaviors. The context for this trial was the management of tendinopathy. The scientific basis for managing tendon injuries has rapidly expanded in recent years [19], and it is also a condition that clinicians encounter regularly.

## Methods

### Design

The study used a mixed-methods approach. The primary outcomes of attitude, knowledge, and behavior change were assessed using a preintervention/postintervention, with qualitative data gathered to contextualize the quantitative results. Ethics approval for this research was obtained through the Monash University Human Research Ethics committee (CF 14/1372-2014000640).

### Participants

The participant group was deliberately inclusive to reflect the nature and reach of social media platforms, and to authentically simulate health professional education via social media. The invitation to participate was distributed via social media—Monash Tendon Research Group social feeds—as well as via an email to the primary clinical affiliates (ie, partner health service sites) of (1) Monash University, Faculty of Medicine, Nursing and Health Sciences, Australia, (2) Monash University Malaysia, (3) Warwick Medical School, University of Warwick, United Kingdom, (4) University of Southern California, and (5) Swami Vivekanand National Institute of Rehabilitation Training and Research, India. Undergraduate students were eligible to participate if they were actively engaged in providing clinical care as part of workplace-based education.

### Intervention

Participants took part in a short course, delivered through the social media platforms of Twitter and Facebook; these were chosen as they are currently the two most widely used social media platforms. The course consisted of eight practice points provided to participants over 2 weeks, with half of the participants randomized to receive the education through Twitter, and half through Facebook. The practice points were developed by tendon researchers, in conjunction with educational and clinical experts. The practice points included "headline" information focused on the key finding or implication for practice, along with supporting materials that typically included a link to a peer-reviewed open access journal article or a podcast, of between 25 and 60 minutes duration, given by clinical experts. Only open access articles were used. Participants were encouraged to utilize the functionality of social media by responding to the practice points with their own comments and opinions, or forwarding messages of interest. Training in participation was provided in the form of a 5-minute video clip, explaining the details of creating an account, and in accessing and following the study’s social media site. The participant would then be able to access the practice points during the course, interpret social media timelines, and contribute to the conversation. A detailed PDF file provided a written version for those who preferred this to video-based instructions.

### Outcomes

Demographic data were collected about participants including country, gender, age, area of practice, years of experience, use of social media, and frequency of contact with people seeking health services for tendon conditions. Kirkpatrick’s hierarchy of educational outcomes proposes that training effects should be examined for four levels of impact: (1) participant reaction, (2) knowledge, (3) change in behavior, and (4) change in health outcomes [20]. Outcomes across levels of impact 1-3 were studied, with the same measures taken at baseline and at 1 week after the intervention.

Participant reactions (Level 1) were measured using the Social Media Use and Perception Instrument (SMUPI). The SMUPI, a questionnaire validated in the population of internal medicine, seeks participant use and attitudes toward social media for continuing professional development [21]. The SMUPI consists of 10 items, each measured across a 5-point Likert scale, with the higher values representing more positive attitudes.

Knowledge (Level 2) was measured via an online examination administered 1 week before and after the short course. The examination included 16 questions; each practice point was allocated two examination questions. The first question related directly to the "headline" information of the practice point, and the second question required knowledge gained from the supporting information provided with the practice point. Questions were in multiple-choice format, with one correct answer and four distractors for each question (see Multimedia Appendix 1). The same questions were used for both baseline and posteducation examinations, except that the questions along with their answers and distractors were randomized to avoid answers based on pattern recognition. Self-rated knowledge was also measured with a 5-point Likert scale (1=very poor, 5=very good) asking the participant to self-rate their perceived knowledge of best practice in tendinopathy management. Self-rated confidence in interacting with people with tendinopathy was also measured using the same 5-point Likert scale.

Changes in behavior (Level 3) were determined by self-reported change in practice, or intended practice, after completing the program. Participants were first asked, “Has the education you have received via social media during this trial changed the way you practice, or intend to practice, with musculoskeletal clients?” followed by the open-text comment, “If you answered ‘yes’ to the question above, please indicate in the space provided below in what way the program has changed your management of tendon clients.” Subsequently, participants had to respond to the following question: “Has the education you have received during this trial increased your use of research evidence within your clinical practice?”

Assessment at all levels of outcomes was administered via an anonymous online survey. Participants entered a unique identifying password that enabled the research team to match presurvey and postsurvey data for each participant to assess change across time. Participants received a certificate of course completion as an incentive, provided after the participant had completed the postcourse examination. To maintain participant anonymity, the mailing address provided for awarding the certificate was not linked to any survey data.

### Data Analysis

Mixed linear models were used to analyze the repeated measurements of the participants. The analyses used all the available data and are based on the assumption that missing values are missing at random. No direct imputation of missing values was undertaken, with the mixed-models approach now regarded as standard [22]. The restricted maximum likelihood (REML) method, as implemented in the GenStat version 17 (VSN International) statistical package [23], was used to fit the models and calculate predicted means; *F* tests were used to test the main effects of time (pre and post). Pairwise least significant difference tests were based on these analyses and conducted at the 5% significance level.  Diagnostic plots of residuals were checked to assess whether or not there were departures from the usual assumptions—homogeneity of variance and normality—required for optimal performance of these statistical tests. Analyses of the 5-point Likert scales also used the same approach as is customary with large datasets [24].  The analyses of binary response outcomes—"changed, or intention to change, practice” and “use of research evidence”—were based on logistic regression models, also fitted using GenStat version 17 (VSN International).

## Results

### Overview

**Figure 1:**
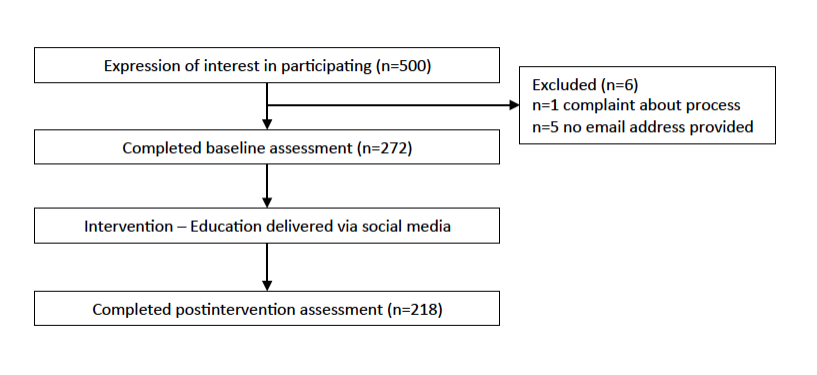
Study flowchart.

Of the 317 total participants who participated in the study's data collection, 99 (31.2%) completed the baseline activities only, 45 (14.2%) completed the postbaseline activities only, and 173 (54.6%) completed the activities at both time points. A study flowchart is provided in Figure 1. Participants represented a spread of professions, ages, and countries, as summarized in Table 1.

**Figure 2:**
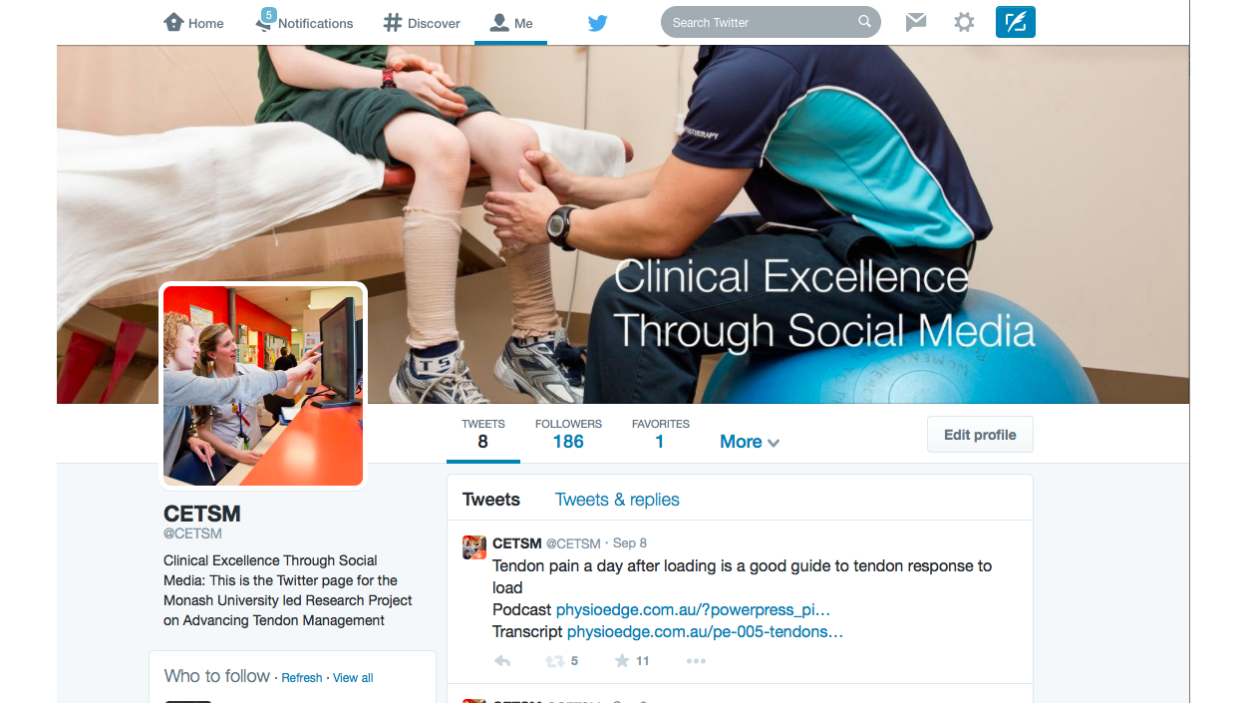
Screenshot of a practice point delivered via Twitter.

**Figure 3:**
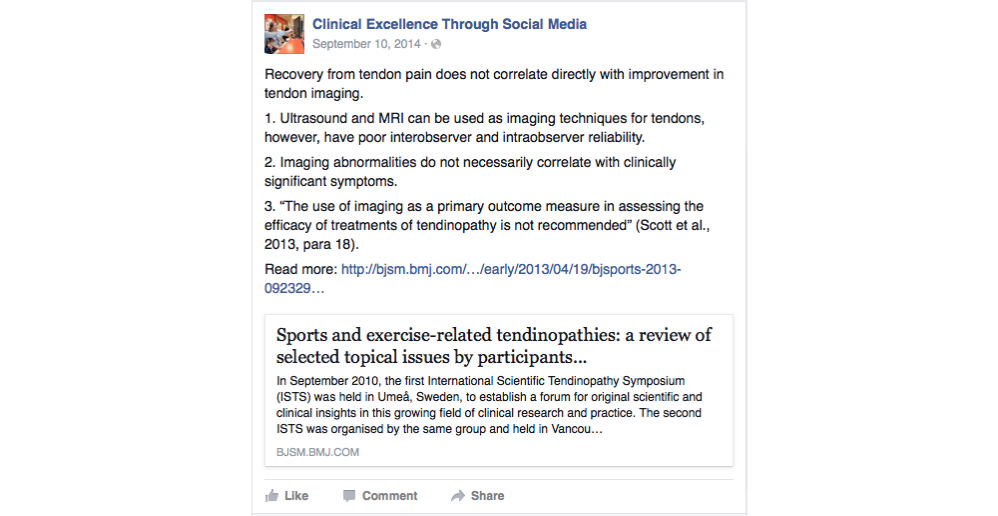
Screenshot of a practice point delivered via Facebook.

The educational program was delivered as described. Screenshots to illustrate the presentation and format of the practice points developed and delivered via Twitter and Facebook are provided in Figures 2 and 3, respectively. The distribution of the eight practice points delivered were spread evenly over the 2-week period.

**Table 1:** Participant demographic data recorded at study baseline.

Descriptor		Participants (n=272), n (%)
**Country**		
	Australia	107 (39.3)
	India	28 (10.3)
	Malaysia	11 (4.0)
	United Kingdom	52 (19.1)
	United States	29 (10.7)
	Other	43 (15.8)
	Not recorded	2 (0.7)
**Age in years**		
	18-24	67 (24.9)
	25-34	123 (45.2)
	35-44	59 (21.7)
	45-54	16 (5.9)
	>54	7 (2.6)
**Profession**		
	Medicine	37 (13.6)
	Physiotherapy	193 (71.0)
	Podiatry	18 (6.6)
	Other	20 (7.4)
	Not specified	4 (1.5)

### Evaluation Outcomes

#### Change in Participant Attitudes to Social Media and Participation (Kirkpatrick’s Level 1)

The participants reported an overall improvement in attitudes toward social media for professional development using total scores for the SMUPI (premean=39.93, pre-SEM=0.41, postmean=41.32, post-SEM= 0.45; SED 0.44, 95% CI 0.57-2.29, *P*=.001), with improved attitudes reported for seven out of the 10 scale items (Table 2). A total of 8.7% (17/195) of participants reported actively engaging with fellow participants via social media during the study period.

**Table 2:** SMUPI^a^ item predicted means from preanalysis/postanalysis.

SMUPI item	Preintervention			Postintervention			SED	95% CI	*P*
	n	Mean	SEM	n	Mean	SEM			
1. I would use social media to gain professional knowledge	269	4.075	0.051	213	4.226	0.056	0.058	0.036-0.266	*.009* ^b^
2. I would use social media to enhance my education or professional development	269	4.084	0.050	212	4.250	0.055	0.057	0.054-0.277	*.003*
3. Social media would be useful for learning about professional development courses	269	4.231	0.048	211	4.268	0.053	0.058	-0.077 to 0.152	.53
4. I would be interested in social media for information about professional development opportunities	269	4.266	0.045	213	4.310	0.050	0.051	-0.057 to 0.145	.35
5. I would like to have professional development courses advertised to me by social media	269	4.047	0.050	212	4.207	0.056	0.060	0.042-0.279	*.007*
6. Professional development courses should use social media to enhance learning	268	3.950	0.052	213	4.095	0.058	0.063	0.020-0.270	*.02*
7. Social media is a professional way to assess professional development course content	269	3.550	0.056	212	3.894	0.063	0.067	0.213-0.476	<*.001*
8. Social media is an ethical way to engage professional development participants	269	3.674	0.053	213	3.972	0.059	0.061	0.178-0.418	<*.001*
9. Social media is an appropriate resource for professional development	269	3.820	0.053	212	4.056	0.058	0.060	0.119-0.354	<*.001*
10. Social media will be increasingly utilized for professional development in the future	268	4.277	0.048	211	4.327	0.054	0.057	-0.063 to 0.163	.42

^a^Social Media Use and Perception Instrument (SMUPI). The scale for all 10 items ranges from 1 (strongly disagree) to 5 (strongly agree).

^b^Statistically significant preanalysis/postanalysis changes are shown in italics.

#### Knowledge Change (Kirkpatrick’s Level 2)

The participant responses to the self-rating of their knowledge of best practice in the management of tendon clients showed a significant increase in Likert scale means pretraining to post-training via the social media intervention (premean = 3.10, postmean = 3.65; difference = 0.55, 95% CI 0.44-0.65; *F*_1,215.7_=103.2, *P*<.001). The participant responses to the self-rating of their confidence for interacting with people with tendinopathy also increased after the social media-based training (premean = 3.30, postmean = 3.71; difference = 0.41, 95% CI 0.31-0.52; *F*_1,211.8_ = 59.32, *P*<.001).

The examination results demonstrated a significant increase in knowledge following the short course (premean = 44.52%, postmean = 61.70%; difference = 17.17%, 95% CI 14.06-20.28; *F*_1,201.4_ = 121.8, *P*<.001). This shift in knowledge was made up of a significant increase in examination questions that were based on the "headline" information within the provided practice points (premean =3.97, postmean = 5.48; difference = 1.51, 95% CI 1.20-1.81; *F*_1,211.4_ = 98.82, *P*<.001), as well as the examination questions based on the further activities (readings or podcasts) provided in the practice points (premean = 3.17, postmean = 4.42; difference = 1.25, 95% CI 0.98-1.53; *F*_1,206.7_ = 80.75, *P*<.001). A subanalysis observed that the significant positive shift in knowledge occurred regardless of profession—medicine, physiotherapy, and podiatry—or country—the United Kingdom, Australia, the United States, Malaysia, and India. A summary table of the pretraining and post-training results for the knowledge change items is provided in Table 3.

**Table 3:** Predicted means for pretraining and post-training knowledge outcomes.

Item	Pretraining			Post-training			SED	95% CI	*P*
	n	Mean or test score	SEM	n	Mean or test score	SEM			
Self-rated knowledge of best practice, mean	270	3.104	0.051	217	3.649	0.056	0.054	0.438-0.651	<.001
Self-rated confidence, mean	271	3.298	0.053	217	3.714	0.058	0.055	0.308-0.524	<.001
Examination (test score), %	259	44.525	1.465	198	61.696	1.629	1.577	14.061-20.282	<.001

#### Behavior Change (Kirkpatrick’s Level 3)

The majority of respondents (136/194, 70.1%) indicated that the education they had received via social media had changed the way they practice, or intended to practice. Those who answered in the affirmative were asked in what way their practice had changed. Thematic analysis of participant open-text responses revealed change in practice concentrated into three key themes: (1) use of evidence-based interventions, (2) patient monitoring, and (3) improved ability for shared (clinician and client) decision making. The following quotes have been provided below to contextualize these themes.

I am more willing to prescribe eccentric exercises and implement the knowledge I learned when treating future patients.

Theme: Evidence-based interventions

She [presenter in podcast] also mentioned using pain ratings after 24hrs post exercise to determine if the load was appropriate. I probably would have responded more to pain during the exercise as an indicator of proper exercise intensity.

Theme: Patient monitoring

The information provided on platelet rich plasma injections would allow me to provide patients with this information in a shared decision-making process.

Theme: Shared decision making

Similarly, a large proportion of respondents (135/193, 69.9%) indicated that the education they had received via social media had increased their use of research evidence within their clinical practice. Thematic analysis of open-text comments to better understand the participant responses revealed that the education provided a motivating reason as to *why* they should improve their evidence-seeking behavior. This largely occurred through surprise at how fast clinically relevant knowledge was advancing.

This course reminded me that even though I am only 2 years out of school, literature is rapidly advancing and changing; therefore it is vital to continue to seek literature to maintain evidence-based practice evidence.

Participant use of research evidence was also influenced through the education by providing the participants with a new method in *how* to seek and access new information.

This was a great tool [social media] to catch up on the literature without having to pay for individual articles and not investing the time into untimely literature searches.

## Discussion

### Principal Findings

This study evaluated the impact of delivering evidence-based knowledge to clinicians via social media. The findings contribute novel information to the field of medical education and evidence-based practice. Under our pretraining/post-training study conditions, clinicians reported positive changes in their attitudes and clinical behaviors, and demonstrated increased knowledge after their social media-based educational intervention. Although this is new empirical knowledge, it aligns with literature evaluating the effectiveness of blended learning approaches involving social media within health professional education [25], and researcher and clinician perspectives on the potential use of social media for effective professional development [10,16,21]. The key contrasting differences between earlier literature investigating knowledge change from social media [15,18,26] is the strength of the methodology and the context of the education focused on translating emerging research evidence into practice.

These preliminary findings, if validated in a randomized and controlled trial, would have implications across the health care, research, and education sectors. From the researcher's perspective, our results may provide new motivation to be outwardly engaged and more connected with clinicians at the coalface. The health researchers and health research centers who have cultivated a large social media following should be encouraged by the findings of this study, which support the potential effectiveness of their efforts. Researchers who are new to social media should be supported to actively disseminate research findings and participate in conversations with clinical stakeholders. From the clinician's perspective, social media is a legitimate form of continuing professional development, with the advantage of being flexible and asynchronous (ie, the material does not have to be accessed at the time of delivery). The extensive global use of social media by clinicians would enable access to minority areas of interest.

From the educator’s perspective, social media provides an opportunity to link health professional learners to new learning opportunities, thereby expanding the classroom and providing a potentially international audience to locally produced education. Social media is most aligned with social-constructivist pedagogy, with the open seeking and sharing of information, encouraging collaboration and cooperation between learners. It may harness the educational advantage of fostering a sense of community for the learner: "Someone is contacting me, and I can talk to them. I can directly contact, and clarify issues with the leaders in the field; my perspectives can influence how experts consider the issues." This is a distinctly new model of student engagement, and a paradigm shift in how we conceptualize education. Education within social media enables a pedagogical approach that appropriately blends academic and personally elating experiences. More than just information seeking, learners have the chance to feel a part of the source of valued information, becoming cocreators and curators of the content. These influences are not limited to undergraduate education, or to the professional development of our existing health workforce industry. Industry requires graduates who have the skills and desire to critically appraise information, improve knowledge, and contribute to practice change [27]. Similarly, learners wish to enter a workforce that is adaptable as opposed to perpetuating outdated practices [17].

A key limitation to the effectiveness of social media for continued professional development, and the translation of new evidence into practice, is that social media relies heavily on the end user to be able to discern relevant information and ascertain whether the information is based on sound evidence. Inability to critique the information presented may result in inaccurate information being created and perpetuated. Dangers also exist for the "education provider" in the social media pathway. Many researchers and clinicians who engage through social media platforms may not be aware of the safe and effective use of the medium, the etiquette of social media, or the application of relevant laws such as copyright and plagiarism. At present, there are limited training opportunities in the professional use of social media for health professionals.

Aside from limitations in the concept of social media for health professional education, limitations also exist within the study conducted that may affect the generalizability of the results. Knowledge change was assessed using the same 16-question multiple-choice exam at baseline and follow-up. This introduces the possibility of a learning effect. To counter this, both the examination questions and response items were presented in a random order. To minimize the risk of participant collaboration between participants during the examination, the learner was reminded that the examination was anonymous (ie, matched by code, rather than participant name) and that correct answers would be provided on completion of the study. There is also potential for reporting bias as participants were asked to self-assess the impact of the learning intervention. This limitation would be addressed in a randomized controlled trial of the intervention, which is recommended as a future line of investigation. A longer follow-up period may allow more detailed data to be collected on behavior change over time. The impact of self-selection and prior social media usage by participants has the potential to affect the participants’ willingness to participate and their reaction to the educational platform.

This work identifies targets for research on social media within health professional education. Researchers, educators, and clinicians cannot ignore the reach of social media in health professional education. This reach is further evidenced by the logistics of this trial, a low-cost approach to bringing together a large number of clinicians from across the world—a research paradigm enabled by the nature of the intervention. Social media has developed into a convenient and acceptable link between clinicians and sources of information for professional development, as well as between researchers and clinicians. Rather than looking at whether or not social media is effective for health professional education, it may be time to look at how various modalities can be optimized, both in terms of how the messages are delivered and how learners can be supported to engage. In addition, the cost-effectiveness and sustainability of such models should be determined to allow informed risk management and to improve the adoption of such modalities. Further research may contribute to existing literature by focusing on the barriers and facilitators for leading researchers and clinicians to transition away from private social media forums to more public forums, allowing them to become more outwardly engaged in reducing the knowledge-to-practice gap in the health professions.

### Conclusions

Social media appears to be an effective educational medium to provide information to clinicians for improving knowledge, fostering the use of research evidence by health professionals, and changing their clinical behaviors by translating new research evidence into clinical practice. Social media equalizes information sharing, thereby challenging historical hierarchies. It enables a direct information-sharing pathway and provides opportunities for discussion among clinicians, health researchers, professional associations, authors of journal articles, and other key industry stakeholders. Using social media to provide tailored and direct connections between researchers and clinicians facilitates the translation of new knowledge and practice, overcoming many of the obstacles within the “evidence pipeline.” In this way, it can help reduce the evidence-to-practice gap—the essence of research translation.

## Appendix

Multimedia Appendix 1 Examination questions used in the assessment of knowledge change.

## Abbreviations

REML: restricted maximum likelihood
SMUPI: Social Media Use and Perception Instrument
