# Supplementary material for: Translating Evidence Into Practice via Social Media: A Mixed-Methods Study
Source: J Med Internet Res. 2015 Oct 26;17(10):e242. doi: 10.2196/jmir.4763 (PMC4642790; doi:10.2196/jmir.4763)
Supplement: Multimedia Appendix 1 [file jmir_v17i10e242_app1.pdf]

Appendix 1. Multiple choice assessment questions
Assessment questions
Correct answers are marked with an asterisk

Question 1

A client presents with chronic mid portion Achilles tendinopathy, and asks for platelet rich plasma (PRP) injections. You could tell them that PRP injections, according to current research evidence, may result in:

a) A better outcome compared to placebo
b) less morning stiffness
c) no difference in outcomes compared to saline injection *
d) Improvement in their tendon structure
e) I don't know

Question 2

After having a platelet rich plasma (PRP) injection for chronic Achilles tendinopathy, the evidence suggests that a person can expect:

a) To be more satisfied with treatment at 24 weeks post injection than if they had received a saline injection
b) That they will be as likely to return to their desired sport at 24 weeks post injection as they would if they had had a saline injection*
c) To have improvements on imaging at 24 weeks compared to those who have had a saline injection
d) To require another PRP injection at 4 weeks
e) I don't know

Question 3

A client presents to you with an Achilles tendinopathy in the reactive stage on the continuum model. The loading intervention most likely to aggravate symptoms is:

a) Reducing current levels of sport or activity
b) A heavy-load eccentric exercise program*
c) Completing non weight bearing exercises (e.g. swimming)
d) Isometric exercises
e) I don't know

Question 4
What loading strategies would be most suitable for a lower limb degenerative tendon?

a) Isometric exercises
b) Eccentric exercises*
c) Swimming
d) Pilates
e) I don't know

Question 5

The following is not a risk factor for tendinopathy:

a) Diabetes
b) High cholesterol
c) Hyperuricaemia
d) Vitamin A deficiency*
e) I don't know

Question 6

Based on current research evidence,which of the following statements istrue?

a) Adiposity is a risk factor for rotator cuff tendinopathy*
b) There is no link between tendinopathies and foot ulcers
c) There is no increased risk for tendon degeneration in people with familial hypercholesterolaemia
d) Individuals with Type 2 Diabetes are not at increased risk of tendon degeneration

e) I don’t know

Question 7

Pain in upper limb tendinopathies is different to lower limb tendinopathies in that:

a) UL tendinopathies are generally more painful
b) Pain reduces more quickly in LL tendinopathies when treated with an eccentric strengthening program
c) UL tendinopathies may be more likely to have a central sensitisation component than LL tendinopathies*
d) The upper limb has more nerve fibres to transmit pain to the central nervous system
e) I don’t know

Question 8

A client presents with chronic patellar tendinopathy with high baseline pain. You prescribe high load isometric exercises. You might see:

a) An immediate reduction in pain*
b) An increase in pain for 2-4 weeks
c) An immediate reduction in swelling
d) An equal effect on the contralateral limb
e) I don’t know

Question 9

You see a client who is completing a strength program of heel raises for Achilles tendinopathy. To determine whether the load was appropriate, when would be the best time to monitor the pain response?

a) during loading
b) immediately after loading
c) 4 hours after loading
d) The next morning*
e) I don’t know

Question 10

A client is referred to you with hamstring tendinopathy. Which exercises would you avoid in the reactive stage?

a) Swimming as a cross training exercise
b) hamstring curls (seated)*
c) Isometric hamstring exercises
d) Bridging exercise
e) I don’t know

Question 11

Which of the following statements is true in relation to the imaging of tendons?

a) An improvement in tendon structure on ultrasound always correlates with improvements in pain and function
b) Imaging of tendons will be able to predict clinical outcomes
c) Ultrasound cannot be used to obtain an image of a tendon
d) A reduction in pain may not correlate with an improvement in tendon structure *
e) I don’t know

Question 12

Which of the following is true regarding tendon imaging?

a) Ultrasound provides more information about tendon structure than MRI*
b) Imaging techniques cannot visualise changes in tendon structure until 6 weeks post initial tendon injury
c) Doppler ultrasound is not used in imaging tendons
d) MRI is the ‘gold standard’ for assessing changes to tendon structure

e) I don’t know

Question 13

Which of the following is true about exercise therapies for tendinopathies?

a) Eccentric exercise is the ‘gold standard’ in exercise therapies for tendinopathies
b) Eccentric exercise may not be beneficial in some tendinopathies*
c) Heavy slow resistance training is not useful in the management of tendinopathies
d) Isometric exercises are appropriate for all stages of tendon management
e) I don’t know

Question 14
Eccentric exercise has been shown to have no effect or worse outcomes in:

a) Mid portion Achilles tendinopathy
b) Insertional Achilles tendinopathy when completed off a step*
c) Lower limb degenerative tendons
d) Rotator cuff tendinopathy
e) I don’t know

Question 15

Tendon pathology may be induced by:
a) Overstretching the tendon
b) Slowly increasing the tensile load on the tendon
c) Inadequate warm up and cool down
d) Compressive loads on the tendon*
e) I don’t know

Question 16
A clinical intervention that would reduce compressive load on the insertion of the Achilles tendon is:

a) A heel raise in the client’s shoe*
b) Increasing the amount of calf stretching
c) Reducing weight bearing exercise
d) Using crutches
e) I don’t know
